# Physical Performance and Activity in Older Prostate Cancer Survivors in Comparison with Population-based Matched Controls

**DOI:** 10.1016/j.euros.2024.11.005

**Published:** 2024-12-10

**Authors:** Reidun Sletten, Marit Slaaen, Line Merethe Oldervoll, Håvard Kjesbu Skjellegrind, Jūratė Šaltytė Benth, Lennart Åstrøm, Øyvind Kirkevold, Sverre Bergh, Bjørn Henning Grønberg, Siri Rostoft, Asta Bye, Paul Jarle Mork, Ola Berger Christiansen

**Affiliations:** a Department of Public Health and Nursing, Faculty of Medicine and Health Sciences, Norwegian University of Science and Technology (NTNU), Trondheim, Norway; b The Research Center for Age-Related Functional Decline and Disease, Innlandet Hospital Trust, Ottestad, Norway; c Department of Oncology and Palliative Care, Innlandet Hospital Trust, Gjøvik/Lillehammer, Norway; d Institute of Clinical Medicine, Faculty of Medicine, University of Oslo, Oslo, Norway; e Centre for Crisis Psychology, Faculty of Psychology, University of Bergen, Bergen, Norway; f The National Institute on Intellectual Disability and Community, Department of Mental Health, Faculty of Medicine and Health Sciences, NTNU, Trondheim, Norway; g HUNT Research Centre, Department of Public Health and Nursing, Faculty of Medicine and Health Sciences, NTNU, Levanger, Norway; h Levanger Hospital, Nord-Trøndelag Hospital Trust, Levanger, Norway; i Institute of Clinical Medicine, Campus Ahus, University of Oslo, Oslo, Norway; j Health Services Research Unit, Akershus University Hospital, Lørenskog, Norway; k Section of Clinical and Experimental Oncology, Department of Immunology, Genetics and Pathology, Uppsala University, Uppsala, Sweden; l Faculty of Health, Care and Nursing, NTNU Gjøvik, Gjøvik, Norway; m The Norwegian National Centre for Ageing and Health, Vestfold Hospital Trust, Tønsberg, Norway; n Department of Clinical and Molecular Medicine, Faculty of Medicine and Health Sciences, NTNU, Trondheim, Norway; o Department of Oncology, St. Olavs Hospital, Trondheim University Hospital, Trondheim, Norway; p Department of Geriatric Medicine, Oslo University Hospital, Oslo, Norway; q Department of Nursing and Health Promotion, Faculty of Health Sciences, Oslo Metropolitan University, Oslo, Norway; r Department of Urology, Innlandet Hospital Trust, Hamar, Norway

**Keywords:** Cancer survivors, Grip strength, Older, One-legged balance, Prostate cancer, Physical activity, Physical performance, Radical treatment, Short Physical Performance Battery

## Abstract

**Background and objective:**

Whether radical prostate cancer treatment affects long-term physical performance and physical activity in older men is not known. We aimed to compare physical performance and self-reported physical activity between relapse-free older prostate cancer survivors and population-based controls.

**Methods:**

A single-centre, cross-sectional study including 109 men aged ≥70 yr receiving robotic-assisted radical prostatectomy (61.5%) or external beam radiotherapy (38.5%) between 2014 and 2018 was conducted. Population-based matched (age, gender, and education) controls (*n* = 327) were drawn from the Trøndelag Health Study. The primary (the Short Physical Performance Battery [SPPB] summary score) and secondary (gait speed, grip strength, one-legged balance, and the self-reported Physical Activity Index) outcomes were compared between survivors and controls by adjusted linear mixed models.

**Key findings and limitations:**

The SPPB score, gait speed, and Physical Activity Index did not differ between survivors (mean age 78.3 yr, mean time since treatment 52.9 mo) and controls (mean age 78.2 yr). Survivors had slightly poorer grip strength (regression coefficient [RC] –5.81, *p* < 0.001, 95% confidence interval [CI] –7.46; –4.17) and one-legged balance (RC –4.36, *p* < 0.001, 95% CI –6.72; –2.00; adjusted models), but the clinical significance is uncertain. Small sample size and potential selection of the fittest survivors are limitations that may reduce the generalisability of our findings.

**Conclusions and clinical implications:**

3 to 8 yr after radical prostate cancer treatment, older men’s overall physical performance and physical activity level were comparable with those of matched controls. This suggests that the treatment had little impact on functional status.

**Patient summary:**

In this study, we investigated physical function in older men several years after they had undergone curatively intended treatment for prostate cancer in comparison with men in a general population of the same age and education. We found that physical function was similar, except slightly poorer grip strength and balance on one leg in men treated for prostate cancer. We conclude that the overall physical function was comparable with that of the general population and believe that this indicates that prostate cancer treatment was well tolerated despite older age.

## Take Home Message

From 3 to 8 yr after radical prostate cancer treatment, older men’s overall physical performance and physical activity level were comparable with those of matched controls. This suggests that the treatment had little impact on functional status.

## 1 Introduction

Prostate cancer is a disease of older age. In western countries, the median age at diagnosis is about 70 yr [1], [2], and with an ageing population, the number of older men with prostate cancer is increasing [3], [4]. Traditionally, older age was a determinant of omitting curative treatment [5], [6], [7], but new guidelines state that overall health rather than chronological age should guide treatment decisions [8], [9], [10].

Although interindividual differences are considerable, ageing is associated with an overall physiological decline [11], [12]. This decline may be accelerated by cancer treatment. Across different cancer diagnoses, reduced physical performance has been observed in older survivors and is associated with poorer survival and a higher risk of disability [13], [14]. To counteract the decline during and after cancer treatment, being physically active is important [15], [16], [17]. Previous studies on physical activity and performance in older prostate cancer survivors describe the negative effects of initiating or maintaining androgen deprivation therapy (ADT; eg, reduced performance and increased fatigue) [18], [19], [20], [21], [22], [23], [24]. Data on long-term status after previous radical prostate cancer treatment, especially in a regular clinical setting and compared with controls, are lacking. Such data are, however, prerequisite to identify rehabilitation needs and guide treatment choices. Moreover, because physical function tends to decline with ageing, a comparison with norm data from men of the same age is paramount to provide a true picture of older men’s physical status after prostate cancer treatment. One Norwegian study found older age to be associated with less frequent physical activity in prostate cancer survivors, but this study did not compare with controls [25]. To the best of our knowledge, only one previous study has compared physical performance in older prostate cancer survivors with population controls, but in this study information about treatment was missing [26]. The few other studies on this topic were either not dedicated to older survivors or lacked a comparison with controls [22], [27], [28], [29].

We have assessed physical performance and level of physical activity in relapse-free older men treated for prostate cancer, and compared these with a matched population-based sample to evaluate long-term treatment effects. Furthermore, we explored whether physical performance was associated with specific side effects from cancer treatment.

## 2 Patients and methods

### 2.1 Setting

The study was conducted at a public hospital with a catchment area of about 370 000 inhabitants . Approximately 300 men receive curative treatment for prostate cancer each year, either robotic-assisted radical prostatectomy (RARP) or external beam radiotherapy (EBRT), the latter in combination with ADT unless contraindicated or refused by the patient.

### 2.2 Study design and participants

**Fig. 1:**
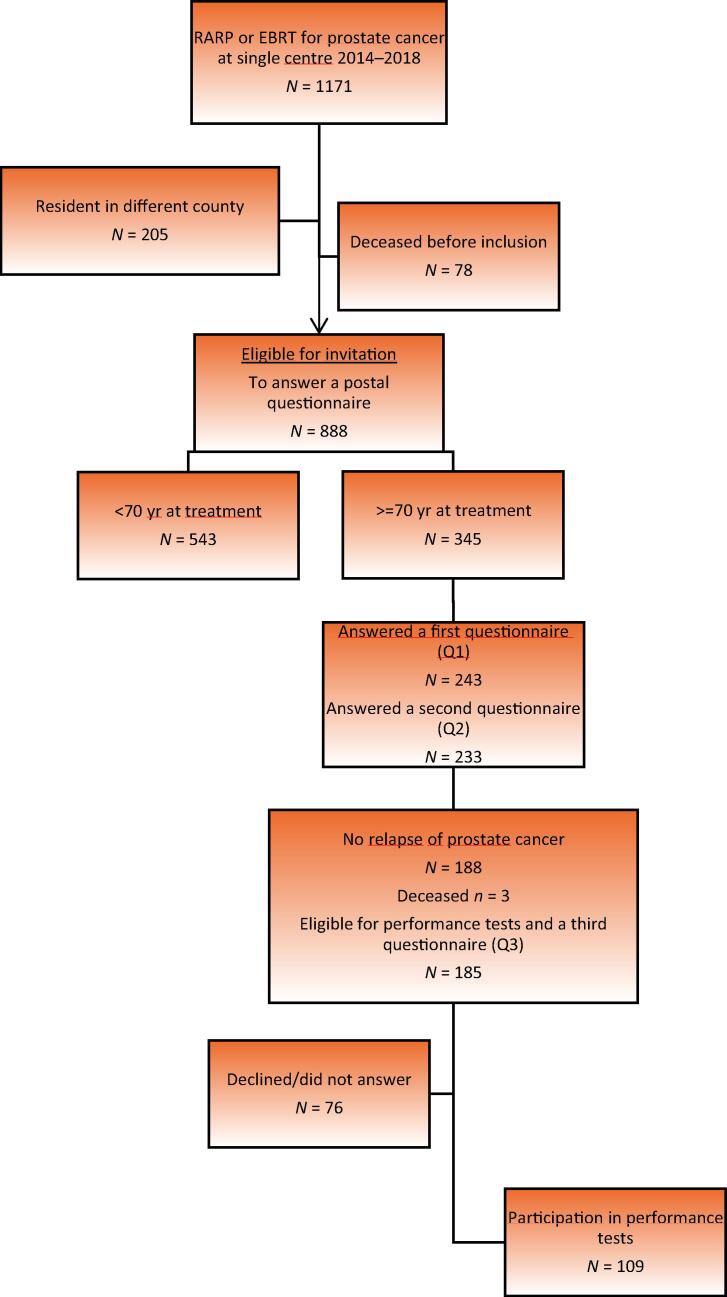
Flow chart showing recruitment of prostate cancer survivors. EBRT = external beam radiotherapy; RARP = robotic-assisted radical prostatectomy.

The study was a part of a larger single-centre cross-sectional project [30]. All Norwegian-speaking residents in the catchment area who received radical treatment for prostate cancer between January 2014 and December 2018 were invited in May 2021 to answer a questionnaire (Q1). Those ≥70 yr of age at the time of prostate cancer treatment received a new questionnaire (Q2) after answering Q1. The eligible cohort for the present study comprised men who answered both Q1 and Q2, and had no clinical relapse of prostate cancer. Clinical relapse was defined as the occurrence of distant metastasis or additional treatment (salvage radiotherapy or surgery, lifelong ADT, chemotherapy, or other medical treatment for recurrent disease). Participants underwent physical performance tests and completed a third questionnaire (Q3; Fig. 1).

Controls (3:1) were from the Trøndelag Health study (HUNT), matching criteria were age at inclusion (±2 yr) and self-reported education, and selection was at random among all possible matches. HUNT is a population-based health survey of the adult population in Trøndelag county, Norway [31], running in several waves from 1984. The fourth wave (HUNT4) conducted in 2017–2019 included 56 042 inhabitants (54% of invited). HUNT4 was the first wave with a substudy dedicated for adults older than 70 yr (HUNT4 70+) and included physical performance tests [31].

For the present study assessments, we used items from the HUNT4 questionnaires and physical performance tests from HUNT4 70+, the European Organisation of Research and Treatment in Cancer Quality of Life Questionnaire-C30 (QLQ-C30) [32], and the Expanded Prostate Cancer Index Composite-26 (EPIC-26) [33], [34].

### 2.3 Study procedures

Eligible survivors were identified from registries in the local hospital. Invitations to participate, Q1 and Q2, and one reminder after 4 wk were sent by postal mail. The men consented to participate by returning the signed consent forms and the completed questionnaires in prepaid return envelopes. Q3 was administered at the test site before performance testing. The performance tests were carried out by a study nurse (cancer nurse) and a PhD student (resident in oncology), who scheduled the tests at a convenient time and location (hospital [*n* = 106] or home [*n* = 3]) for the participants through a telephone call.

### 2.4 Outcomes and outcome assessment

The primary outcome was the Short Physical Performance Battery (SPPB) summary score. The secondary outcomes were hand grip strength, one-legged balance, gait speed, and self-reported physical activity summary index (Physical Activity Index). In addition, physical function (QLQ-C30) was used as an outcome in explorative analyses.

The SPPB assesses lower extremity function by testing standing balance on two legs (side by side, semitandem, and tandem), gait speed (time completing 4 m walking course), and ability to rise from a chair [35]. Each test is scored on a scale of 0–4 (0 indicating not able to and 4 indicating the best performance) with a maximum sum score of 12.

Grip strength (kg) was tested with the Jamar hand dynamometer, in a sitting position. Maximal grip was held for 1–2 s and tested three times for each hand. The maximum value was used, irrespective of the dominant hand.

One-legged balance (in seconds) tested static balance ability [36] by asking participants to stand on their leg of choice, for a maximum of 30 s with eyes open.

Gait speed (in metres per second) was derived from the 4 m gait speed test included in the SPPB, carried out at participants’ normal, comfortable walking speed.

The Physical Activity Index ranges from 0 to 15 and was calculated from three sets of questions, including average frequency, intensity, and duration of weekly exercises [37]. A high score means a higher level of physical activity.

The items in the physical function domain of the QLQ-C30 [32] are answered on a 4-point Likert scale ranging from 1 (not at all) to 4 (very much). For the analysis, the physical function scores were transformed to scales ranging from 0 to 100, with a higher score meaning better function. A difference in mean scores of ≥10 is considered clinically significant [38].

### 2.5 Other assessments

These included the following:

1. Sociodemographic measures, self-reported, including cohabitant status (living alone or not) and education (primary school/high school/vocational education/college or university).
2. Comorbidities, self-reported, registered as “1 = yes” or “0 = no” on 18 medical conditions (summary score 0–18) similar to those in HUNT4, except that “cancer” was changed to “cancer other than prostate cancer”.
3. Charlson Comorbidity Index (CCI) without age adjustment, based on information retrieved by manual search in the hospital electronic medical records, estimated for the survivors and used for an explorative analysis [39]. Information to calculate CCI for controls was unavailable.
4. Treatment-related side effects, self-reported on the EPIC-26 covering five domains, of which three (urinary incontinence, urinary irritative/obstructive, and bowel) were used in the present analyses [33], [34]. The scores on each item were standardised and transformed linearly to a scale from 0 to 100. Higher scores mean more symptoms.

### 2.6 Ethics

The project was approved by Regional Committee for Medical Research Ethics Southeast Norway (REK South East; ID 266644) and Innlandet Hospital Trust’s official data protection officer, and registered in ClinicalTrials.gov (NCT05200039). All participants provided written informed consent.

### 2.7 Statistical analysis

Descriptive characteristics were reported as means, standard deviations (SDs), minimum and maximum values, or frequencies and percentages, as appropriate. Sociodemographic characteristics and comorbidities were compared between the prostate cancer survivors and controls, and the responders and nonresponders, by independent sample *t* test or χ^2^ test. In addition, self-reported general health from an earlier study part was compared between responders and nonresponders.

The primary and secondary outcomes were compared between cancer survivors and controls by linear mixed models adjusting for within-pair correlations. The models were adjusted for cohabitant status and self-reported comorbidities. Sensitivity analyses stratified by treatment modality (EBRT and RARP) were performed to account for the potential effect of differences in treatment, which for EBRT included ADT for most.

To explore whether treatment-related side effects (EPIC-26) were associated with physical function, linear regression analyses among prostate cancer survivors were performed with the SPPB summary score, gait speed, and physical function domain of the QLQ-C30 as outcomes. We estimated one model for each outcome, with one variable on side effects at a time. The analyses were adjusted for CCI, age, and treatment modality.

Regression analyses were performed on survivors and controls with no missing values on confounders. To assess the potential bias, the excluded and included survivors/controls were compared by independent sample *t* test with respect to age, comorbidities, sociodemographic characteristics, and outcome variables.

## 3 Results

A total of 185 eligible men were invited to participate and 109 responded (58.9%; Fig. 1). More responders had undergone RARP (61.5%) than nonresponders (32.9%), and responders reported better general health than nonresponders (Table 1).

**Table 1:** Comparison of descriptive statistics and self-reported general health of responders and nonresponders

Variable	Nonresponders(*n* = 76)	Responders(*n* = 109)	*p* value
Age, mean (SD)	78.8 (3.0)	78.3 (3.2)	0.29 a
Educational attainment, *n* (%)			
Primary school	17 (23.6)	21 (19.4)	0.09 b
High school	22 (30.1)	24 (22.2)	
Vocational education	18 (25.0)	21 (19.4)	
College/university	15 (20.8)	42 (38.9)	
*n*	72	108	
Primary treatment, *n* (%)			
RARP	25 (32.9)	67 (61.5)	<0.001 b,*
EBRT	51 (67.1)	42 (38.5)	
*n*	76	109	
Months since treatment, mean (SD)	57.3 (18.1)	52.9 (16.6)	0.09 a
CCI, mean (SD)	1.3 (1.4)	1.0 (1.3)	0.08 a
General health (from Q1), *n* (%)			
Poor	1 (1.4)	5 (4.6)	0.01 c,*
Not so good	27 (38.6)	18 (16.7)	
Good	35 (50.8)	72 (66.7)	
Very good	7 (10.0)	13 (12.0)	
*n*	70	108	

CCI = Charlson Comorbidity Index; EBRT = external beam radiotherapy; RARP = robotic-assisted radical prostatectomy; SD = standard deviation.

* Significance level *p* < 0.05.

a Two-sided independent sample *t* test, equal variances assumed.

b Chi-square test.

c Fisher exact test.

A total of 327 matched controls were drawn randomly. The mean (SD) age at inclusion was 78.3 (3.2) yr for the survivors and 78.2 (3.2) yr for the controls (Table 2). Only the mean sum score of self-reported comorbidities was slightly lower for survivors than for controls (1.2 vs 1.6, *p* = 0.011), with no other differences. Of the survivors, 67 (61.5%) had received RARP as primary treatment, while 42 (38.5%) received EBRT. Most (90.5%) of those treated with EBRT had ADT as part of the initial treatment. The mean (SD) time since treatment was 52.9 (16.6) mo.

**Table 2:** Descriptive statistics for survivors and controls

Variable	Controls (*n* = 327)	Survivors (*n*= 109)	*p* value
Age			
Min, max	72.8, 88.2	72.9, 86.9	
Mean (SD)	78.2 (3.2)	78.3 (3.2)	0.350 a
Educational attainment (*n* = 327 + 108), *n* (%)			
Primary school	63 (19.3)	21 (19.4)	0.999 b
High school	75 (22.9)	24 (22.2)	
Vocational education	63 (19.3)	21 (19.4)	
College/university	126 (38.5)	42 (38.9)	
Cohabitant status (*n* = 325 + 109), *n* (%)			
Living alone	63 (19.4)	18 (16.5)	0.496 c
Living with others	262 (80.6)	91 (83.5)	
Comorbidities (self-reported; *n* = 277 + 107)			
Min, max	0, 8	0, 7	
Mean (SD)	1.6 (1.5)	1.2 (1.4)	0.011
CCI (*n* = 109)			
Min, max	NA	1.0 (1.3)	a,*
Mean (SD)	NA	0, 6	
Primary treatment (*n* = 109), *n* (%)			
RARP	NA	67 (61.5)	NA
EBRT	NA	42 (38.5)	
Adjuvant ADT	NA	38 (90.5 d)	NA

ADT = androgen deprivation therapy; CCI = Charlson Comorbidity Index; EBRT = external beam radiotherapy; NA = not applicable; RARP = robotic-assisted radical prostatectomy; SD = standard deviation.

* Significance level *p* < 0.05.

a *p* value from linear mixed model adjusting for within-pair correlations.

b *p*-value χ^2^ test.

c *p* value from hierarchical multinomial regression model adjusting for within-pair correlations.

d Percentage of EBRT.

Outcomes are presented in Table 3. The mean (SD) SPPB summary scores were 10.5 (2.0) for survivors and 10.6 (1.8) for controls, and 44% versus 42.8% had a maximum score of 12 points.

**Table 3:** Descriptive statistics for outcome variables

Outcome	Controls (*n* = 327)	Survivors (*n* = 109)
*Primary outcome, mean (SD)*		
SPPB summary score	10.6 (1.8)	10.5 (2.0)
*Secondary outcomes, mean (SD)*		
Grip strength (kg; *n* = 325 + 109)	43.5 (9.4)	38.6 (7.2)
One-legged balance (s; *n* = 324 + 101)	14.6 (11.2)	11.8 (10.1)
Gait speed (m/s; *n* = 326 + 109)	1.0 (0.3)	1.0 (0.2)
Physical Activity Index (*n* = 318 + 107)	3.5 (3.0)	3.4 (3.1)
*Explorative outcome, mean (SD)*		
Physical function (QLQ-C30; *n* = 0 + 107)	NA	85.9 (17.6)

NA = not applicable; QLQ-C30 = European Organisation of Research and Treatment in Cancer Quality of Life Questionnaire-C30; SD = standard deviation; SPPB = Short Physical Performance Battery.

Numbers are means (standard deviations [SDs]) if not otherwise specified.

Two (1.8%) survivors and 52 (15.9%) controls had missing data on covariates and were excluded from the regression models. Excluded survivors/controls had a lower SPPB summary score (10.8 [SD 1.6] vs 9.5 [SD 2.9], *p* < 0.001), shorter one-legged balance time (14.4 [SD 11.1] vs 10.9 [SD 10.4], *p* = 0.031), and lower gait speed (1.0 [SD 0.2] vs 0.9 [SD 0.3], *p* = 0.028) than those included in the analysis. Neither unadjusted nor adjusted models showed any significant differences between survivors and controls for the SPPB score, gait speed, or Physical Activity Index (Table 4). Unadjusted models showed a 5.47 kg lower grip strength in survivors than in controls (regression coefficient [RC] –5.47, *p* < 0.001 and 95% confidence interval [95% CI] –7.06; –3.88), and 3.49 s shorter one-legged balance time (RC –3.49, *p* = 0.004, 95% CI –5.86; –1.13). In adjusted models, survivors had 5.81 kg lower grip strength (RC –5.81, *p* < 0.001, 95% CI –7.46; –4.17) and 4.36 s shorter one-legged balance time (RC –4.36, *p* < 0.001, 95% CI –6.72; –2.00) than controls (Table 4). Sensitivity analyses, stratified by the treatment modalities EBRT and RARP, showed similar results to the main analyses, with the only exception that among EBRT strata, there were no longer any differences between survivors and controls with respect to one-legged balance (Supplementary Tables 1 and 2).

**Table 4:** Results of linear mixed model comparing controls and cancer survivors for primary and secondary outcomes, unadjusted and adjusted analyses

	Unadjusted model		Adjusted model	
	RC (95% CI)	*p* value	RC (95% CI)	*p* value
*SPPB summary score* (*n* = 382)				
Control—ref. Survivor	0 –0.31 (–0.71; 0.09)	0.132	0 –0.38 (–0.77; 0.01)	0.057
Cohabitant status, living with others a Comorbidities a	0.78 (0.22; 1.35) –0.11 (–0.22; –0.007)	0.006 0.037	0.78 (0.21; 1.35) –0.11 (–0.22; –0.01)	0.007 0.033
*Grip strength (kg; n* = *381)*				
Control—ref. Survivor	0 –5.47 (–7.06; –3.88)	<0.001	0 –5.81 (–7.46; –4.17)	<0.001 *
Cohabitant status, living with others a Comorbidities a	1.06 (–1.00; 3.12) –0.75 (–1.31; –0.18)	0.312 0.009	1.17 (–0.88; 3.21) –0.76 (–1.32; –0.21)	0.264 0.007
*One-legged balance (s; n* = *372)*				
Control—ref. Survivor	0 –3.49 (–5.86; –1.13)	0.004	0 –4.36 (–6.72; –2.00)	<0.001 *
Cohabitant status, living with others a Comorbidities a	2.97 (–0.02; 5.96) –1.49 (–2.15; –0.83)	0.051 <0.001	3.03 (0.01; 6.05) –1.50 (–2.14; –0.86)	0.049 <0.001
*Gait speed (m/s; n* = *382)*				
Control—ref. Survivor	0 –0.02 (–0.07; 0.02)	0.284	0 –0.04 (–0.08; 0.008)	0.106
Cohabitant status, living with others a Comorbidities a	0.10 (0.04; 0.16) –0.02 (–0.03; –0.007)	0.001 0.003	0.10 (0.04; 0.16) –0.02 (–0.03; –0.008)	0.002 0.002
*Physical Activity Index (n* = *375)*				
Control—ref. Survivor	0 –0.05 (–0.66; 0.56)	0.867	0 –0.14 (–0.75; 0.47)	0.648
Cohabitant status, living with others a Comorbidities a	0.48 (–0.26; 1.22) –0.15 (–0.36; 0.06)	0.205 0.165	0.49 (–0.26; 1.25) –0.15 (–0.36; 0.06)	0.198 0.161

CI = confidence interval; RC = regression coefficient; ref. = reference; SPPB = Short Physical Performance Battery.

Due to missing values on several confounders, the number of survivors and controls with results available for analysis (*n*) was lower than expected.

* Significance level *p* < 0.05.

a Pair (control vs case) variable is present in these models.

Explorative analyses showed no associations between side effects and physical performance, except for urinary incontinence and gait speed (RC 0.002, 95% CI 0.0; 0.003, *p* = 0.037; Supplementary Table 1).

## 4 Discussion

We investigated physical performance and self-reported physical activity in older prostate cancer survivors compared with matched population controls. There was no difference in the SPPB summary score, gait speed, or Physical Activity Index. However, the survivors had poorer performance in grip strength and one-legged balance.

Despite a broad focus on physical function in relation to ADT, little is known regarding older prostate cancer survivors’ long-term physical performance after radical treatment , compared with other older men. The only study we are aware of used the 400 m walk test and the “Health ABC physical performance battery” in 117 older prostate cancer survivors matched with 468 controls [26]. The results are in line with our findings on the SPPB and gait speed, showing no inferior function in cancer survivors. In contrast to our study, however, no information about cancer stage or treatment was recorded, and the survivors performed better than controls on grip strength. Another study, including smaller groups of older and younger survivors and healthy controls, showed similar results [27]. Neither of these studies reported results for one-legged balance test.

An SPPB summary score of >10, combined with a gait speed of approximately 1.0 m/s and a Physical Activity Index of >=3.4, indicates good functional status [40], [41] and a relatively high level of self-reported physical activity in both survivors and controls [42]. Supported by the results of others [26], [27], these findings suggest that radical prostate cancer treatment had little impact on long-term physical performance. The survivors’ performance on grip strength and one-legged balance was also good, although slightly poorer than controls. The mean grip strength (38.6 kg) was well above the cut-offs for weak (26 kg) and intermediate (32 kg) grip strength in men [43], and one-legged balance was better than in the 19 participants in the only other study we have found reporting such results in older prostate cancer survivors [22].

The clinical significance of the differences in favour of controls, 5.81 kg for grip strength, and 4.36 s for one-legged balance can thus be questioned. The minimal clinically important difference (MCID) for grip strength is reportedly 5.0–6.5 kg, but it is not clear whether this is valid when grip strength is normal [44]. The MCID for the balance test is scarcely investigated [45]. However, increments in grip strength as small as 5 kg is associated with all-cause mortality in patients with a previous diagnosis of cancer [14], and the ability to complete the 10-s one-legged balance is associated with all-cause mortality in general [46]. Poorer results on these tests might indicate increased vulnerability and a potential unmet need of rehabilitation after treatment. Owing to a ceiling effect, with >40% having the highest possible score, the SPPB may lack sufficient sensitivity to detect differences in highly functioning individuals. Still, arbitrary gait speed was also similar between groups, indicating comparable mobility.

As it is likely that only fitter older men are selected for radical treatment, one might hypothesise that our study participants should have performed better than controls. This was not the case, and due to the lack of pretreatment information, post-treatment deterioration from a higher level of functioning cannot be ruled out. Still, our results showed that in a selected group of men, physical performance is good despite radical prostate cancer treatment in older age, and there is no clinically relevant difference in performance from that of matched controls. The results indicate that older men seem to tolerate radical treatment well, and this should be emphasised in clinical decision-making. The findings need confirmation in larger studies in the future.

The only statistically significant association found in explorative analyses, between gait speed and urinary incontinence, was minor (RC 0.002). Even if we thought it plausible, mainly from clinical experience, that treatment-related side effects could affect older men’s physical performance through reduced ability to maintain physical activity [25], [47], we did not find support for this in our explorative analyses in prostate cancer survivors only. Owing to the explorative design and small sample size, this statistically significant finding must be interpreted with caution.

### 4.1 Strengths and limitations

The main study strengths are the use of validated instruments, objective testing of physical performance using several measures, and a comparison with matched controls, drawn from a population considered to be representative of Norwegians [31]. Moreover, comprehensive information about nonresponding prostate cancer survivors from earlier study parts allowed for a proper evaluation of the representability of the responders.

Several limitations must be considered. First, several factors affect study generalisability. In line with the inclusion criteria, the results are applicable only to older men living without a clinical relapse after radical treatment for prostate cancer. Being a single-centre study, it is possible that our cohort might not be representative of those selected for radical treatment in other centres or health care systems. Moreover, the sample size was small, and the responding prostate cancer survivors reported somewhat better health than nonresponders, indicating a selection of the fitter part of the eligible population. Second, the cross-sectional design does not allow for any determination of causality between treatment and present performance, or evaluate development over time. The time elapsed between treatment and performance assessments also varied, that is, from 3 to 8 yr. For all survivors, it was however >2 yr, which in our experience means that any change related to prostate cancer treatment had stabilised. Third, survivors and controls excluded from regression analyses had a lower SPPB summary score and gait speed, and shorter one-legged balance time. As more controls than survivors were excluded, this may have affected results primarily by overestimating the difference between the two groups. Finally, the small sample size implicated that subgroup analyses are questionable and hampered by low statistical power. For these reasons as well as variation in duration and time since termination of ADT, we could not evaluate the specific contribution of previous adjuvant ADT. Sensitivity analyses stratified by treatment modality (EBRT and RARP) had results similar to the main analyses, but due to a small group size, these must be interpreted with caution.

## 5 Conclusions

In our study on men ≥70 yr of age at radical treatment for prostate cancer, we found that physical performance and activity levels at a mean of 52.9 mo after treatment were comparable with those in matched controls. Grip strength and one-legged balance were slightly poorer in prostate cancer survivors than in controls, but the clinical significance of the differences is uncertain.

  ***Author contributions*:** Reidun Sletten had full access to all the data in the study and takes responsibility for the integrity of the data and the accuracy of the data analysis.

  *Study concept and design*: All authors.

*Acquisition of data*: Sletten, Christiansen, Slaaen.

*Analysis and interpretation of data*: All authors.

*Drafting of the manuscript*: Sletten, Christiansen, Oldervoll, Slaaen.

*Critical revision of the manuscript for important intellectual content*: All authors.

*Statistical analysis*: Benth, Sletten.

*Obtaining funding*: Slaaen.

*Administrative, technical, or material support*: None.

*Supervision*: Slaaen, Christiansen.

*Other*: None.

  ***Financial disclosures:*** Reidun Sletten certifies that all conflicts of interest, including specific financial interests and relationships and affiliations relevant to the subject matter or materials discussed in the manuscript (eg, employment/affiliation, grants or funding, consultancies, honoraria, stock ownership or options, expert testimony, royalties, or patents filed, received, or pending), are the following: None.

  ***Funding/Support and role of the sponsor*:** This work was supported by Innlandet Hospital Trust, under Grant 150410.

  ***Acknowledgements*:** The Trøndelag Health Study (HUNT) is a collaboration between HUNT Research Centre (Faculty of Medicine and Health Sciences, Norwegian University of Science and Technology NTNU), Trøndelag County Council, Central Norway Regional Health Authority, and the Norwegian Institute of Public Health. The authors are thankful to all former patients who participated in this study, and to study nurses Bodil Sem Kolsgaard and Anna Enger for their contribution in data collection. We would also like to thank our user participants Leif Henning Asla, John Jørgen Renberg, and Tore Sørum, who have participated in the design of the study and discussion of results.

  ***Data sharing*:** Owing to a statement by the Data Protection Officer at Innlandet Hospital Trust, and in accordance with Norwegian privacy regulations, data cannot be shared publicly because these are confidential (due to the consent given by the men when included in the study). It is possible to extract information, upon request. Proposals should be directed to the Research Department of Innlandet Hospital Trust (contact: SIHFDLforskning@sikt.sykehuspartner.no).
