# Corrigendum to “Physical Performance and Activity in Older Prostate Cancer Survivors in Comparison with Population-based Matched Controls” [Eur. Urol. Open Sci. 71 (2025) 85–95]

**DOI:** 10.1016/j.euros.2025.05.005

**Published:** 2025-06-16

**Authors:** Reidun Sletten, Marit Slaaen, Line Merethe Oldervoll, Håvard Kjesbu Skjellegrind, Jūratė Šaltytė Benth, Lennart Åstrøm, Øyvind Kirkevold, Sverre Bergh, Bjørn Henning Grønberg, Siri Rostoft, Asta Bye, Paul Jarle Mork, Ola Berger Christiansen

**Affiliations:** a Department of Public Health and Nursing, Norwegian University of Science and Technology (NTNU), Trondheim, Norway; b The Research Center for Age-Related Functional Decline and Disease, Innlandet Hospital Trust, Ottestad, Norway; c Department of Oncology and Palliative Care, Innlandet Hospital Trust, Gjøvik/Lillehammer, Norway; d Institute of Clinical Medicine, Faculty of Medicine, University of Oslo, Oslo, Norway; e Faculty of Medicine and Health Sciences, Department of Mental Health, The National Institute on Intellectual Disability and Community, NTNU, Trondheim, Norway; f Centre for Crisis Psychology, Faculty of Psychology, University of Bergen, Bergen, Norway; g HUNT Research Centre, Department of Public Health and Nursing, Faculty of Medicine and Health Sciences, NTNU, Levanger, Norway; h Levanger Hospital, Nord-Trøndelag Hospital Trust, Levanger, Norway; i Institute of Clinical Medicine, Campus Ahus, University of Oslo, Oslo, Norway; j Health Services Research Unit, Akershus University Hospital, Lørenskog, Norway; k Section of Clinical and Experimental Oncology, Department of Immunology, Genetics and Pathology, Uppsala University, Uppsala, Sweden; l Faculty of Health, Care and Nursing, NTNU Gjøvik, Gjøvik, Norway; m The Norwegian National Centre for Ageing and Health, Vestfold Hospital Trust, Tønsberg, Norway; n Department of Clinical and Molecular Medicine, NTNU, Trondheim, Norway; o Department of Oncology, St. Olavs Hospital, Trondheim University Hospital, Trondheim, Norway; p Department of Geriatric Medicine, Oslo University Hospital, Oslo, Norway; q Oslo Metropolitan University, Oslo, Norway; r Department of Urology, Innlandet Hospital Trust, Hamar, Norway

On February 21, 2025, the HUNT Study’s team of researcher’s informed us of an error in their data on grip strength in HUNT participants. The error resulted in a deviation of 1 to 9 kg grip strength, compared to the original data. In response to the error, the team provided us with a new dataset containing updated values for the variable for our matched population-based controls. To determine whether this error affected our published results (Sletten et al. European Urology Open Science, 2025 [accepted for publication Nov. 2024]), we reran our analyses for grip strength with unadjusted and adjusted linear mixed models to compare cancer survivors and controls. We found that the differential directionality between survivors and matched controls remained unchanged and as previously published, with slightly poorer grip strength among survivors. The RC, however, was less in the updated calculations than the RC initially reported. Furthermore, with respect to sensitivity analyses (i.e., separate analyses for survivors having received RARP and survivors having received EBRT, respectively), the difference between survivors and controls was statistically significant in the RARP subgroup only, that is, there was no longer any significant difference between survivors and controls in the EBRT subgroup (see Tables A1, A2, AS1 and AS2).

Because the differences in grip strength identified in the published analyses were slight and of uncertain clinical significance (*Eur Urol Open Sci 2025 Vol. 71, p 93*), we find that the revealed error does not affect our conclusions.

**Table A1**. Results from updated dataset provided from the HUNT Study’s team of researchers. Descriptive statistics for outcome variables. Numbers are means (standard deviations (SDs)) if not otherwise specified. Values that deviate from the originally published values in bold italic.

**Table:** 

Outcome	Controls (n = 327)	Survivors (n = 109)
***Primary outcome, mean (SD)***		
SPPB summary score	10.6 (1.8)	10.5 (2.0)
***Secondary outcomes, mean (SD)***		
***Grip strength (kg) (n = 325 + 109)***	***41.8 (9.0)***	38.6 (7.2)
One-legged balance (sec) (n = 324+101)	14.6 (11.2)	11.8 (10.1)
Gait speed (m/s) (n = 326+109)	1.0 (0.3)	1.0 (0.2)
Physical Activity Index (n = 318+107)	3.5 (3.0)	3.4 (3.1)
***Explorative outcome, mean (SD)***		
Physical function (QLQ-C30) (n = 0+107)	NA	85.9 (17.6)

**Table A2**. Results from updated dataset provided from the HUNT Study’s team of researchers. Linear mixed model comparing controls and cancer survivors for primary and secondary outcomes, unadjusted and adjusted analyses (RC=regression coefficient; CI=confidence interval). Values that deviate from the originally published values in bold italic.

**Table:** 

	Unadjusted model		Adjusted model	
	RC (95% CI)	p-value	RC (95% CI)	p-value
***SPPB summary score* (n = 382)**				
Control – ref.	0		0	
Survivor	−0.31 (−0.71; 0.09)	0.132	−0.38 (−0.77; 0.01)	0.057
Cohabitant status, Living with others^1^	0.78 (0.22; 1.35)	0.006	0.78 (0.21; 1.35)	0.007
Comorbidities^1^	−0.11 (−0.22; −0.007)	0.037	−0.11 (−0.22; −0.01)	0.033
***Grip strength (kg)* (n = 381)**				
Control – ref.	0		0	
Survivor	***−3.79 (−5.35;−2.23)***	<0.001	***−4.11 (−5.70;−2.51)***	<0.001*
Cohabitant status, Living with others^1^	*1.24 (*−*0.77; 3.26)*	*0.277*	*1.33 (−0.68; 3.35)*	*0.195*
Comorbidities^1^	*−0.67 (−1.22; −0.12)*	*0.017*	*−0.68 (−1.22; −0.14)*	*0.013*
***One-legged balance (s)* (n = 372)**				
Control – ref.	0		0	
Survivor	−3.49 (−5.86; −1.13)	0.004	−4.36 (−6.72; −2.00)	<0.001*
Cohabitant status, Living with others^1^	2.97 (−0.02; 5.96)	0.051	3.03 (0.01; 6.05)	0.049
Comorbidities^1^	−1.49 (−2.15; −0.83)	<0.001	−1.50 (−2.14; −0.86)	<0.001
***Gait speed (m/s)* (n = 382)**				
Control – ref.	0		0	
Survivor	−0.02 (−0.07; 0.02)	0.284	−0.04 (−0.08; 0.008)	0.106
Cohabitant status, Living with others^1^	0.10 (0.04; 0.16)	0.001	0.10 (0.04; 0.16)	0.002
Comorbidities^1^	−0.02 (−0.03; −0.007)	0.003	−0.02 (−0.03; −0.008)	0.002
***Physical Activity Index* (n = 375)**				
Control – ref.	0		0	
Survivor	−0.05 (−0.66; 0.56)	0.867	−0.14 (−0.75; 0.47)	0.648
Cohabitant status, Living with others^1^	0.48 (−0.26; 1.22)	0.205	0.49 (−0.26; 1.25)	0.198
Comorbidities^1^	−0.15 (−0.36; 0.06)	0.165	−0.15 (−0.36; 0.06)	0.161

Due to missing values on several confounders, the number of survivors and controls with results available for analysis (n) was lower than expected.

^1^Pair (control vs. case) variable is present in these models.

RC = regression coefficient; CI = confidence interval; SPPB = short physical performance battery; PA Index = physical activity index.

*significance level p <0.05.

**Table AS1 Strata RARP**. Results from updated dataset provided from the HUNT Study’s team of researchers. Linear mixed model comparing cancer survivors previously treated with RARP with their matched controls for primary and secondary outcomes, unadjusted and adjusted analyses (RC=regression coefficient; CI=confidence interval). Updated dataset Values that deviate from the originally published values in bold italic.

**Table:** 

	Unadjusted model		Adjusted model	
	RC (95% CI)	p-value	RC (95% CI)	p-value
***SPPB summary score* (n = 236)**				
Control – ref.	0		0	
Survivor	−0.10 (−0.55; 0.35)	0.669	−0.16 (−0.59; 0.27)	0.471
Cohabitant status, Living with others*	0.99 (0.18; 1.80)	0.017	0.98 (0.16; 1.79)	0.019
Comorbidities*	−0.08 (−0.22; 0.05)	0.214	−0.07 (−0.20; 0.05)	0.238
***Grip strength (kg)* (n = 235)**				
Control – ref.	***0***		***0***	
Survivor	***−4.72 (−6.49;−2.95)***	***<0.001***	***−5.23 (−7.06;−3.40)***	***<0.001***
Cohabitant status, Living with others*	***0.04 (−2.14; 2.32)***	***0.937***	***0.13 (***−***2.00; 2.26)***	***0.904***
Comorbidities*	***−0.77 (−1.39−0.16)***	***0.013***	***−0.77 (−1.39;−0.16)***	***0.013***
***One-legged balance (s)* (n = 232)**				
Control – ref.	0		0	
Survivor	−4.20 (−7.25; −1.14)	0.007	−5.28 (−8.42; −2.14)	0.001
Cohabitant status, Living with others*	1.32 (−1.89; 5.53)	0.539	1.22 (−2.76; 5.20)	0.548
Comorbidities*	−1.45 (−2.39; −0.51)	0.002	−1.45 (−2.36; −0.54)	0.002
***Gait speed (m/s)* (n = 236)**				
Control – ref.	0		0	
Survivor	−0.02 (−0.08; 0.04)	0.439	−0.04 (−0.10; 0.01)	0.136
Cohabitant status, Living with others*	0.15 (0.06; 0.23)	<0.001	0.14 (0.06; 0.23)	0.001
Comorbidities*	−0.03 (−0.04; −0.009)	0.003	−0.03 (−0.04; −0.009)	0.002
***Physical Activity Index* (n = 233)**				
Control – ref.	0		0	
Survivor	0.14 (−0.59; 0.87)	0.712	−0.08 (−0.84; 0.68)	0.843
Cohabitant status, Living with others*	0.39 (−0.68; 1.46)	0.474	0.37 (−0.70; 1.44)	0.500
Comorbidities*	−0.30 (−0.52; −0.09)	0.006	−0.30 (−0.52; −0.08)	0.007

*Pair (control vs. case) variable is present in these models.

**Table AS2 strata EBRT**. Results from updated dataset provided from the HUNT Study’s team of researchers. Linear mixed model comparing cancer survivors previously treated with EBRT with their matched controls for primary and secondary outcomes, unadjusted and adjusted analyses (RC = regression coefficient; CI = confidence interval). Values that deviate from the originally published values in bold italic.

**Table:** 

	Unadjusted model		Adjusted model	
	RC (95% CI)	p-value	RC (95% CI)	p-value
***SPPB summary score* (n = 146)**				
Control – ref.	0		0	
Survivor	−0.67 (−1.42; 0.08)	0.081	−0.70 (−1.44; 0.04)	0.065
Cohabitant status, Living with others*	0.46 (−0.29; 1.20)	0.229	0.48 (−0.27; 1.24)	0.211
Comorbidities*	−0.11 (−0.29; 0.06)	0.198	−0.12 (−0.29; 0.05)	0.167
***Grip strength (kg)* (n = 146)**				
Control – ref.	***0***		***0***	
Survivor	***−2.40 (−5.24;−0.44)***	***0.097***	***−2.57 (−5.37; 0.22)***	***0.071***
Cohabitant status, Living with others*	***2.98 (***−***0.23; 6.18)***	***0.069***	***3.05 (***−***0.21; 6.32)***	***0.067***
Comorbidities*	***−0.46 (−1.41; 0.49)***	***0.340***	***−0.49 (−1.42; 0.45)***	***0.306***
***One-legged balance (s)* (n = 140)**				
Control – ref.	0		0	
Survivor	−2.52 (−6.20; 1.17)	0.181	−2.96 (−6.74; 0.82)	0.125
Cohabitant status, Living with others*	4.69 (1.15; 8.23)	0.009	4.82 (1.13; 8.51)	0.010
Comorbidities*	−1.50 (−2.41; −0.60)	0.001	−1.54 (−2.42; −0.66)	0.001
***Gait speed (m/s)* (n = 146)**				
Control – ref.	0		0	
Survivor	−0.03 (−0.10; 0.04)	0.425	−0.03 (−0.10; 0.04)	0.401
Cohabitant status, Living with others*	0.02 (−0.06; 0.10)	0.585	0.02 (−0.06; 0.10)	0.545
Comorbidities*	−0.008 (−0.03; 0.01)	0.380	−0.009 (−0.03; 0.009)	0.347
***Physical Activity Index* (n = 142)**				
Control – ref.	0		0	
Survivor	−0.40 (−1.49; 0.69)	0.471	−0.44 (−1.56; 0.68)	0.440
Cohabitant status, Living with others*	0.60 (−0.38; 1.58)	0.228	0.58 (−0.40; 1.55)	0.249
Comorbidities*	0.11 (−0.28; 0.50)	0.576	0.10 (−0.30; 0.50)	0.619

*Pair (control vs. case) variable is present in these model.

