# Supplementary material for: Physical Performance and Activity in Older Prostate Cancer Survivors in Comparison with Population-based Matched Controls
Source: Eur Urol Open Sci. 2024 Dec 10;71:87–95. doi: 10.1016/j.euros.2024.11.005 (PMC11697607; doi:10.1016/j.euros.2024.11.005)
Supplement: Supplementary Data 1 [file mmc1.docx]

# Supplementary tables

### Sensitivity analyses

**Supplementary Table 1. Strata RARP**. Results of linear mixed model comparing cancer survivors previously treated with RARP with their matched controls for primary and secondary outcomes, unadjusted and adjusted analyses (RC=regression coefficient; CI=confidence interval)

|  | Unadjusted model | | Adjusted model | |
| --- | --- | --- | --- | --- |
|  | RC (95% CI) | p-value | RC (95% CI) | p-value |
| ***SPPB summary score* (n=236)** | | | | |
| Control – ref.  Case | 0  -0.10 (-0.55; 0.35) | 0.669 | 0  -0.16 (-0.59; 0.27) | 0.471 |
| Cohabitant status, Living with others*  Comorbidities* | 0.99 (0.18; 1.80)  -0.08 (-0.22; 0.05) | 0.017  0.214 | 0.98 (0.16; 1.79)  -0.07 (-0.20; 0.05) | 0.019  0.238 |
| ***Grip strength (kg)* (n=235)** | | | | |
| Control – ref.  Case | 0  -6.22 (-8.03; -4.40) | <0.001 | 0  -6.77 (-8.69; -4.86) | <0.001 |
| Cohabitant status, Living with others*  Comorbidities* | 0.44 (-1.99; 2.87)  -0.83 (-1.47; -0.20) | 0.722  0.010 | 0.50 (-1.81; 2.82)  -0.83 (-1.46; -0.21) | 0.669  0.009 |
| ***One-legged balance (s)* (n=232)** | | | | |
| Control – ref.  Case | 0  -4.20 (-7.25; -1.14) | 0.007 | 0  -5.28 (-8.42; -2.14) | 0.001 |
| Cohabitant status, Living with others*  Comorbidities* | 1.32 (-1.89; 5.53)  -1.45 (-2.39; -0.51) | 0.539  0.002 | 1.22 (-2.76; 5.20)  -1.45 (-2.36; -0.54) | 0.548  0.002 |
| ***Gait speed (m/s)* (n=236)** | | | | |
| Control – ref.  Case | 0  -0.02 (-0.08; 0.04) | 0.439 | 0  -0.04 (-0.10; 0.01) | 0.136 |
| Cohabitant status, Living with others*  Comorbidities* | 0.15 (0.06; 0.23)  -0.03 (-0.04; -0.009) | <0.001  0.003 | 0.14 (0.06; 0.23)  -0.03 (-0.04; -0.009) | 0.001  0.002 |
| ***Physical Activity Index* (n=233)** | | | | |
| Control – ref.  Case | 0  0.14 (-0.59; 0.87) | 0.712 | 0  -0.08 (-0.84; 0.68) | 0.843 |
| Cohabitant status, Living with others*  Comorbidities* | 0.39 (-0.68; 1.46)  -0.30 (-0.52; -0.09) | 0.474  0.006 | 0.37 (-0.70; 1.44)  -0.30 (-0.52; -0.08) | 0.500  0.007 |

*Pair (control vs. case) variable is present in these models

**Supplementary Table 2. Strata EBRT**. Results of linear mixed model comparing cancer survivors previously treated with EBRT with their matched controls for primary and secondary outcomes, unadjusted and adjusted analyses (RC=regression coefficient; CI=confidence interval)

|  | Unadjusted model | | Adjusted model | |
| --- | --- | --- | --- | --- |
|  | RC (95% CI) | p-value | RC (95% CI) | p-value |
| ***SPPB summary score* (n=146)** | | | | |
| Control – ref.  Case | 0  -0.67 (-1.42; 0.08) | 0.081 | 0  -0.70 (-1.44; 0.04) | 0.065 |
| Cohabitant status, Living with others*  Comorbidities* | 0.46 (-0.29; 1.20)  -0.11 (-0.29; 0.06) | 0.229  0.198 | 0.48 (-0.27; 1.24)  -0.12 (-0.29; 0.05) | 0.211  0.167 |
| ***Grip strength (kg)* (n=146)** | | | | |
| Control – ref.  Case | 0  -4.38 (-7.28; -1.48) | 0.003 | 0  -4.51 (-7.43; -1.59) | 0.002 |
| Cohabitant status, Living with others*  Comorbidities* | 2.15 (-1.19; 5.49)  -0.58 (-1.56; 0.39) | 0.207  0.241 | 2.25 (-0.93; 5.43)  -0.60 (-1.58; 0.37) | 0.165  0.226 |
| ***One-legged balance (s)* (n=140)** | | | | |
| Control – ref.  Case | 0  -2.52 (-6.20; 1.17) | 0.181 | 0  -2.96 (-6.74; 0.82) | 0.125 |
| Cohabitant status, Living with others*  Comorbidities* | 4.69 (1.15; 8.23)  -1.50 (-2.41; -0.60) | 0.009  0.001 | 4.82 (1.13; 8.51)  -1.54 (-2.42; -0.66) | 0.010  0.001 |
| ***Gait speed (m/s)* (n=146)** | | | | |
| Control – ref.  Case | 0  -0.03 (-0.10; 0.04) | 0.425 | 0  -0.03 (-0.10; 0.04) | 0.401 |
| Cohabitant status, Living with others*  Comorbidities* | 0.02 (-0.06; 0.10)  -0.008 (-0.03; 0.01) | 0.585  0.380 | 0.02 (-0.06; 0.10)  -0.009 (-0.03; 0.009) | 0.545  0.347 |
| ***Physical Activity Index* (n=142)** | | | | |
| Control – ref.  Case | 0  -0.40 (-1.49; 0.69) | 0.471 | 0  -0.44 (-1.56; 0.68) | 0.440 |
| Cohabitant status, Living with others*  Comorbidities* | 0.60 (-0.38; 1.58)  0.11 (-0.28; 0.50) | 0.228  0.576 | 0.58 (-0.40; 1.55)  0.10 (-0.30; 0.50) | 0.249  0.619 |

*Pair (control vs. case) variable is present in these model

#### Explorative analyses

**Supplementary Table 3**. Results of linear regression model for association between **SPPB** **summary score** as outcome and EPIC-26 variables, unadjusted and adjusted analyses (RC=regression coefficient; CI=confidence interval) among survivors only

|  | Unadjusted model | | Adjusted model | |
| --- | --- | --- | --- | --- |
|  | RC (95% CI) | p-value | RC (95% CI) | p-value |
| Urinary incontinence (n=98)  CCI  Age at inclusion  Primary treatment, EBRT | 0.01 (-0.01; 0.03)  -0.26 (-0.52; 0.009)  -0.10 (-0.20; -0.01)  -0.86 (-1.59; -0.12) | 0.262  0.058  0.028  0.024 | 0.02 (-0.003; 0.04)  -0.24 (-0.49; 0.005)  -0.04 (-0.17; 0.09)  -0.72 (-1.69; 0.24) | 0.093  0.054  0.555  0.139 |
| Urinary irritative (n=105)  CCI  Age at inclusion  Primary treatment, EBRT | 0.02 (-0.007; 0.04)  -0.34 (-0.68; -0.001)  -0.11 (-0.21; -0.02)  -0.72 (-1.48; 0.03) | 0.161  0.049  0.024  0.060 | 0.009 (-0.02; 0.03)  -0.31 (-0.66; 0.03)  -0.06 (-0.20; 0.08)  -0.38 (-1.45; 0.69) | 0.510  0.076  0.397  0.483 |
| Bowel (n=103)  CCI  Age at inclusion  Primary treatment, EBRT | 0.007 (-0.02; 0.03)  -0.32 (-0.70; 0.05)  -0.11 (-0.21; -0.02)  -0.75 (-1.52; 0.02) | 0.599  0.089  0.019  0.055 | 0.0001 (-0.03; 0.03)  -0.28 (-0.67; 0.10)  -0.06 (-0.19; 0.08)  -0.49 (-1.54; 0.57) | 0.996  0.146  0.416  0.360 |

**Supplementary Table 4**. Results of linear regression model for association between **Gait speed** as outcome and EPIC-26 variables, unadjusted and adjusted analyses (RC=regression coefficient; CI=confidence interval) among survivors only

|  | Unadjusted model | | Adjusted model | |
| --- | --- | --- | --- | --- |
|  | RC (95% CI) | p-value | RC (95% CI) | p-value |
| Urinary incontinence (n=98)  CCI  Age at inclusion  Primary treatment, EBRT | 0.001 (-0.0003;0.003)  -0.02 (-0.04; 0.004)  -0.009 (-0.02; 0.001)  -0.07 (-0.15; 0.004) | 0.125  0.102  0.082  0.064 | 0.002 (0.0001; 0.003)  -0.02 (-0.05; 0.007)  -0.004 (-0.02; 0.009)  -0.06 (-0.16; 0.04) | 0.037  0.144  0.546  0.235 |
| Urinary irritative (n=105)  CCI  Age at inclusion  Primary treatment, EBRT | 0.002 (-0.001; 0.005)  -0.03 (-0.06; -0.001)  -0.01 (-0.02; -0.002)  -0.06 (-0.14; 0.01) | 0.178  0.042  0.019  0.095 | 0.001 (-0.002; 0.004)  -0.03 (-0.06; 0.003)  -0.009 (-0.02; 0.005)  -0.02 (-0.12; 0.09) | 0.425  0.071  0.208  0.735 |
| Bowel (n=103)  CCI  Age at inclusion  Primary treatment, EBRT | 0.0001 (-0.002;0.002)  -0.04 (-0.07; -0.002)  -0.01 (-0.02; -0.002)  -0.08 (-0.15; -0.002) | 0.916  0.039  0.017  0.044 | -0.0006 (-0.003;0.001)  -0.03 (-0.07; 0.003)  -0.007 (-0.02; 0.007)  -0.05 (-0.15; 0.05) | 0.554  0.077  0.306  0.332 |

**Supplementary Table 5**. Results of linear regression model for association between **Physical function (QLQ-C30)** as outcome and EPIC-26 variables, unadjusted and adjusted analyses (RC=regression coefficient; CI=confidence interval) among survivors only

|  | Unadjusted model | | Adjusted model | |
| --- | --- | --- | --- | --- |
|  | RC (95% CI) | p-value | RC (95% CI) | p-value |
| Urinary incontinence (n=96)  CCI  Age at inclusion  Primary treatment, EBRT | 0.06 (-0.14; 0.26)  -4.34 (-8.32; -0.37)  -0.12 (-1.12; 0.88)  -5.18 (-12.18; 1.81) | 0.529  0.032  0.818  0.145 | 0.13 (-0.07; 0.33)  -4.57 (-8.48; -0.66)  0.62 (-0.61; 1.85)  -6.45 (-14.29; 1.39) | 0.211  0.023  0.320  0.106 |
| Urinary irritative (n=103)  CCI  Age at inclusion  Primary treatment, EBRT | 0.29 (-0.04; 0.63)  -5.21 (-9.26; -1.16)  -0.18 (-1.19; 0.84)  -3.53 (-10.65; 3.59) | 0.080  0.012  0.729  0.328 | 0.30 (-0.006; 0.60)  -5.29 (-9.26; -1.31)  0.45 (-0.80; 1.71)  -1.33 (-8.99; 6.33) | 0.055  0.010  0.477  0.731 |
| Bowel (n=101)  CCI  Age at inclusion  Primary treatment, EBRT | 0.19 (-0.05; 0.44)  -4.79 (-9.04; -0.53)  -0.52 (-1.42; 0.38)  -5.17 (-12.26; -1.91) | 0.113  0.028  0.256  0.151 | 0.15 (-0.13; 0.42)  -4.47 (-9.02; -0.11)  0.10 (-1.11; 1.32)  -3.46 (-11.46; 4.55) | 0.299  0.045  0.867  0.393 |
